# miR-7 methylation as a biomarker to predict poor survival in early-stage non-small cell lung cancer patients

**DOI:** 10.1186/s13578-019-0326-7

**Published:** 2019-08-07

**Authors:** Carlos Rodríguez-Antolín, Laura Felguera-Selas, Olga Pernía, Olga Vera, Isabel Esteban, Itsaso Losantos García, Javier de Castro, Rocío Rosas-Alonso, Inmaculada Ibanez de Caceres

**Affiliations:** 1 Cancer Epigenetics Laboratory, INGEMM, La Paz University Hospital, Madrid, Spain; 2 Biomarkers and Experimental Therapeutics in Cancer, IdiPAZ, Madrid, Spain; 3 Department of Pathology, La Paz University Hospital, Madrid, Spain

## Abstract

Non-small-cell lung cancer (NSCLC) is the most common malignancy worldwide. Platinum-based chemotherapy is the standard of care for these patients. Recent research showed that miR-7 methylation status is a biomarker of cisplatin resistance in lung and ovarian cancer cells, which is one of the major limitations associated with their clinical management. The aim of the present study is to provide clinical insights associated with this novel potential biomarker in NSCLC patients by comparing the miR-7 methylation status with the cisplatin treatment response. Our results analyzed in 81 samples show that miR-7 methylation is a common event in tumor tissue and it is more frequent as the stage of the disease advances, remaining in 75% of metastatic patients. Tumor miR-7 unmethylation trend to a better PFS in early stages, and when our data was validated in an extended *“*in silico” cohort of 969 patients we obtained a significant increment in PFS and OS in those patients harboring miR-7 unmethylated (p = 0.010 and p = 0.007 respectively). When we select those early-stages patients harbouring miR-7 methylation, we observed that adenocarcinoma patients present a dramatic decrease in PFS compared with squamous cell carcinoma patients (median 18.9 versus 59.7 months, p = 0.002). In conclusion, our results show that presence of miR-7 methylation in early-stage NSCLC is suggestive of aggressive behavior, especially for adenocarcinoma patients. One major challenge in early diagnosis in NSCLC is identify the subgroup of patients that could benefit for adjuvant therapy, our data establish the basis for epigenetic classification on early-stage NSCLC that could influence treatment decisions in the future.

**Electronic supplementary material:**

The online version of this article (10.1186/s13578-019-0326-7) contains supplementary material, which is available to authorized users.

Dear Editor,

Lung cancer is the leading cause of cancer-related deaths and represents approximately half of the economic burden of respiratory diseases worldwide. It has been estimated that there were 224,210 new cases of lung cancer (116,000 in men and 108,210 in women) and 159,260 deaths in the United States in 2014 [1]. Non-small-cell lung cancer (NSCLC) represents approximately 80% to 85% of all lung cancers. At the time of diagnosis approximately 70% of NSCLC patients already have advanced or metastatic disease not amenable to surgical resection [2]. Furthermore, 20–40% of early-stage NSCLC patients develop recurrence after a complete resection [3]. Platinum-based combination chemotherapy is the standard of care for the majority of these patients with median overall survival of 10 to 12 months [2]. In the age of targeted therapies, few patients can benefit with this type of treatment and there is an extreme need to investigate novel biomarkers that can help clinical management and response outcomes.

In articles recently published by our group [4, 5], miR-7 hypermethylation was described as a cisplatin resistance biomarker in ovarian and lung cancer cells. These data were validated in ovarian cancer patients, showing a lower overall survival and a premature recurrence. The experimental results strongly support the direct regulation of MAFG through miR-7 as a mechanism involved in detoxification protecting cells from the oxidative stress caused by cisplatin. Based on the relevance and novelty of this study and taking into account that cisplatin is the first line treatment not only for many ovarian cancer patients but also for many lung cancer patients, our aim was to validate these results in NSCLC patients.

We analyzed miR-7 methylation status in 81 Formalin-Fixed Paraffin embedded (FFPE) NSCLC samples, 42 early stages (I, II, IIIA), 31 advanced stages (IIIB, IIIC, IV) and 8 samples obtained from pulmonary biopsies with nonneoplastic lung pathology were used as control tissues. Samples were obtained from three of the main hospitals biobanks in Spain, La Paz University Hospital, Hospital del Mar, and CIBERES (PMC cohort). Inclusion criteria included patients with NSCLC, tumor tissue available in biobank and informed consent signed according to the ethical committee (PI-2109).

The DNA from all the samples was isolated using QIAamp DNA FFPE Tissue Kit (QIAGEN), modified by sodium bisulfite and pre-amplified with primer forward 5′TTAGGAAGAAGTTAGGAGGGGAAA-3′ and primer reverse 5′-CRCTATCCRAATATTTAATACT-3′. Finally, methylation status was obtained by quantitative Methylation-Specific PCR (qMSP) using two Taqman probes, one specifically for methylated CpG positions and another for the unmethylated ones, making it possible to measure the percentage of methylated DNA in a unique PCR reaction. We used the primer/probe set for methylated reaction: F:5′-GGGTGGGGTTTTTTAAGAATC-3′; R: 5′-ACATTCTCCTCCTTCGATCG-3′; Probe: 5′-FAM-ACCCCTCTTCGTTCTCGAT-3′) and for unmethylation F: 5′-GGGGTGGGGTTTTTTAAGAATT-3′; R:5′-ATAACATTCTCCTCCTTCAATCA-3′; Probe: 5′-VIC-ACCCCTCTTCATTCTCAAT-3′). The percentage of methylation of each sample was calculated as 100/(1 + (2^(Ct FAM-Ct VIC))) [6]. Example of methylated and unmethylated qMSP amplification is shown in Additional file 1: Figure S1. Values were considered positive for methylation when ≥ 10% of DNA molecules analyzed were positive.

We used a second independent cohort of 969 patients from The Cancer Genome Atlas (TCGA) database for validation. For miR-7 methylation we interrogated the genomic position (g. 4769531, GRCh37) included in the TCGA methylation arrays. This position was one of the interrogated by the qMSP technology developed by our group and therefore we are analyzing the same methylation effect in both cohorts (“*in house*” and TCGA). 914 of the 969 tumors were early-stages (446 adenocarcinomas and 468 squamous), 55 were advanced stages (29 adenocarcinomas and 26 squamous). Association between miR-7 and patient characteristics were assessed using a Chi squared, Student’s t-test and Mann–Whitney U test. The survival data was calculated by using the Cox proportional hazard model, Kaplan–Meier and Log Rank test. Statistical analysis was performed using SAS 9.3 (SAS Institute, Cary, NC, USA).

We found in the PMC cohort that 25% of non-tumor samples, 64% of tumor early-stage samples and 74% of tumor advanced stage samples were methylated for miR-7, with a significant difference between groups (Fig. 1). From the TCGA cohort, 66% of patients with early-stage harbored methylated miR-7 and 75% in advanced stage. Therefore, we found that methylation signatures obtained from our PMC cohort were equivalent with those defined in TCGA cohorts (64% vs 66% in early-stages and 74% vs 75% in advanced stages, respectively). These outcomes showed that miR-7 methylation was present in nonneoplastic tissues and that it occurred more frequently in disseminated disease.

**Fig. 1:**
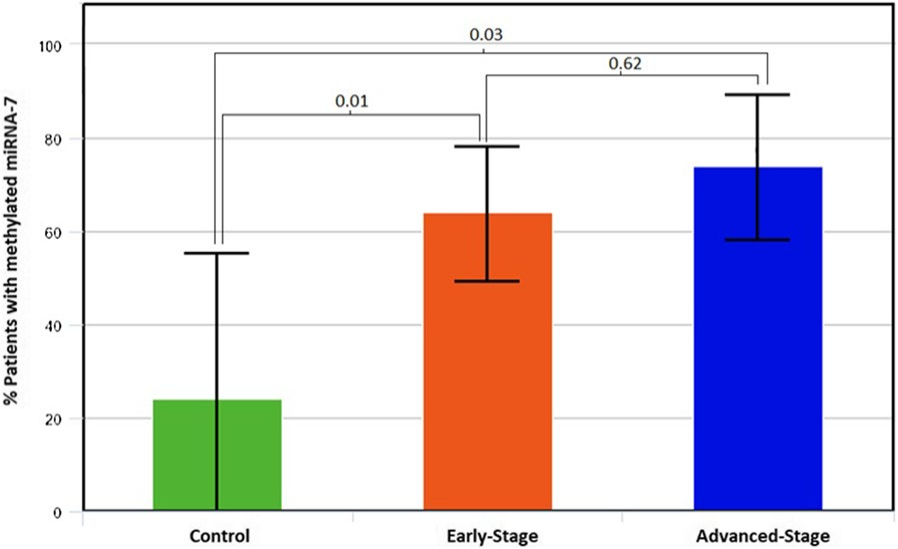
Percentage of patients harboring miR-7 methylation in control, early and advanced-stages. MiR-7 methylation levels were measured by qMSP. To obtain the percentage of methylation for each sample the following equation was used: ${\text{Cmeth}}\, = \,{{100} \mathord{\left/ {\vphantom {{100} {\left[ { 1\, + \, 2^{{\left( {{\text{CT}}_{\text{CG}} - {\text{CT}}_{\text{TG}} } \right)}} } \right]}}} \right. \kern-0pt} {\left[ { 1\, + \, 2^{{\left( {{\text{CT}}_{\text{CG}} - {\text{CT}}_{\text{TG}} } \right)}} } \right]}}$. We showed a statistical significant difference among lung cancer patients and non-cancer controls

The demographic characteristics of the PMC patients are shown in Table 1 and there were not association with miR-7 methylation, probably due to the sample size limit. In the TCGA cohorts, the methylation rates differ significantly by sex (p < 0.01), histology type (p < 0.01) and smoking status (p < 0.01), being miR-7 methylation more common among females, adenocarcinomas and never smokers (Table 2 and Fig. 3a). This data suggest that miR-7 methylation is an event probably more related with tumor development than smoking status.

**Table 1:** Demographic and clinical characteristics of early and advanced-stages NSCLC patients in PMC cohort

	N = 42	Methylated	Unmethylated	p-value
PMC cohort: early-stage				
Sex				
Female	6	2 (33%)	4 (67%)	1.00
Male	36	13 (36%)	23 (64%)	
Smoking status				
Current smoker	25	9 (36%)	16 (64%)	0.88
Former smoker	13	5 (38%)	8 (62%)	
Never smoker	4	1 (25%)	3 (75%)	
Tumor histologic type				
Adenocarcinoma	24	10 (42%)	14 (58%)	n.a
Squamous	16	3 (19%)	13 (81%)	
Others	2	2 (100%)	0 (0%)	
Stage				
IA	2	0 (0%)	2 (100%)	n.a
IB	11	1 (9%)	10 (91%)	
IIA	3	0 (0%)	3 (100%)	
IIB	10	7 (70%)	3 (30%)	
IIIA	16	7 (44%)	9 (56%)	

	N = 31	Methylated	Unmethylated	p-value
PMC cohort: advanced-stage				
Sex				
Female	8	6 (75%)	2 (25%)	0.67
Male	23	17 (74%)	6 (26%)	
Smoking status				
Current smoker	11	8 (73%)	3 (27%)	1.00
Former smoker	20	15 (75%)	5 (25%)	
Tumor histologic type				
Adenocarcinoma	15	12 (80%)	3 (20%)	n.a
Squamous	14	10 (71%)	4 (29%)	
Others	2	1 (50%)	1 (50%)	
Stage				
IIIB	15	11 (73%)	4 (27%)	n.a
IIIC	2	1 (50%)	1 (50%)	
IV	14	11 (79%)	3 (21%)	
RECIST				
PR	10	7 (70%)	3 (30%)	n.a
SD	7	5 (71%)	2 (29%)	
PD	6	6 (100%)	0 (0%)	
n.a.	8			

MiR-7 methylation related to gender, smoking status, tumor histologic type, stage and RECIST. It is not possible to calculate p value because of the number of cases in each group is insufficient

*PR* partial response, *SD* stable disease, *PD* disease progression, *n.a.* not available

**Table 2:** Demographic and clinical characteristics of early and advanced-stages NSCLC patients in TCGA database cohort

	N = 914	Methylated	Unmethylated	p-value
TCGA cohort: early-stage				
Sex				
Female	372	295 (79%)	77 (21%)	< 0.01
Male	542	307 (57%)	235 (43%)	
Smoking status				
Current smoker	809	510 (63%)	299 (37%)	< 0.01
Never smoker	83	77 (93%)	6 (7%)	
n.a.	22			
Tumor histologic type				
Adenocarcinoma	446	423 (95%)	23 (5%)	< 0.01
Squamous	468	179 (38%)	289 (62%)	
Stage				
IA	221	156 (71%)	65 (29%)	< 0.045
IB	278	187 (67%)	91 (33%)	
IIA	113	63 (56%)	50 (44%)	
IIB	160	98 (61%)	62 (39%)	
IIIA	130	90 (69%)	40 (31%)	
n.a.	12			

	N = 55	Methylated	Unmethylated	p-value
TCGA cohort: advanced-stage				
Sex				
Female	17	15 (88%)	2 (12%)	0.12
Male	38	26 (68%)	12 (32%)	
Smoking status				
Current smoker	45	32 (70%)	13 (30%)	0.10
Never smoker	7	0 (0%)	7 (100%)	
n.a.	3			
Tumor histologic type				
Adenocarcinoma	29	27 (93%)	2 (7%)	0.01
Squamous	26	14 (54%)	12 (46%)	
Stage				
IIIB	26	17 (65%)	9 (35%)	0.14
IV	29	24 (83%)	5 (17%)	

MiR-7 methylation related to gender, smoking status, tumor histologic type, stage and RECIST

*n.a.* result not available in TCGA cohort

We then analyze patient’s survival in relation to miR-7 methylation by using Kaplan–Meier survival curves. Our results performed in 42 samples showed that the progression-free survival (PFS) was better in the NSCLC early-stage patients harboring an unmethylated miR-7, although there was no statistically significance (Fig. 2a), probably due to a matter of samples size. In fact, when we performed the same analysis using a high dataset comprising 879 early-stage samples obtained from the TCGA dataset, we obtained enough statistical power. The median PFS in the TCGA cohort was significantly shorter for patients with methylated miR-7 tumors (24.2 months; 95% confidence interval [CI] 18.5 to 29.8), than for patients with unmethylated miR-7 tumors (48.5 months; 95% CI 24.3 to 72.7), corresponding to a hazard ratio (HR) for disease progression of 0.69 (95% CI 0.52 to 0.92; p-value = 0.010). The 5-year progression-free survival rate was 44.5% in patients with unmethylated miR-7 versus 33.0% with methylated (Fig. 2b). The median overall survival (OS) was not reached in unmethylated group versus 41.2 months (95% CI 34.0 to 48.4) in methylated group (HR 0.664; 95% CI 0.494 to 0.894; p-value = 0.007) (Fig. 2c).

**Fig. 2:**
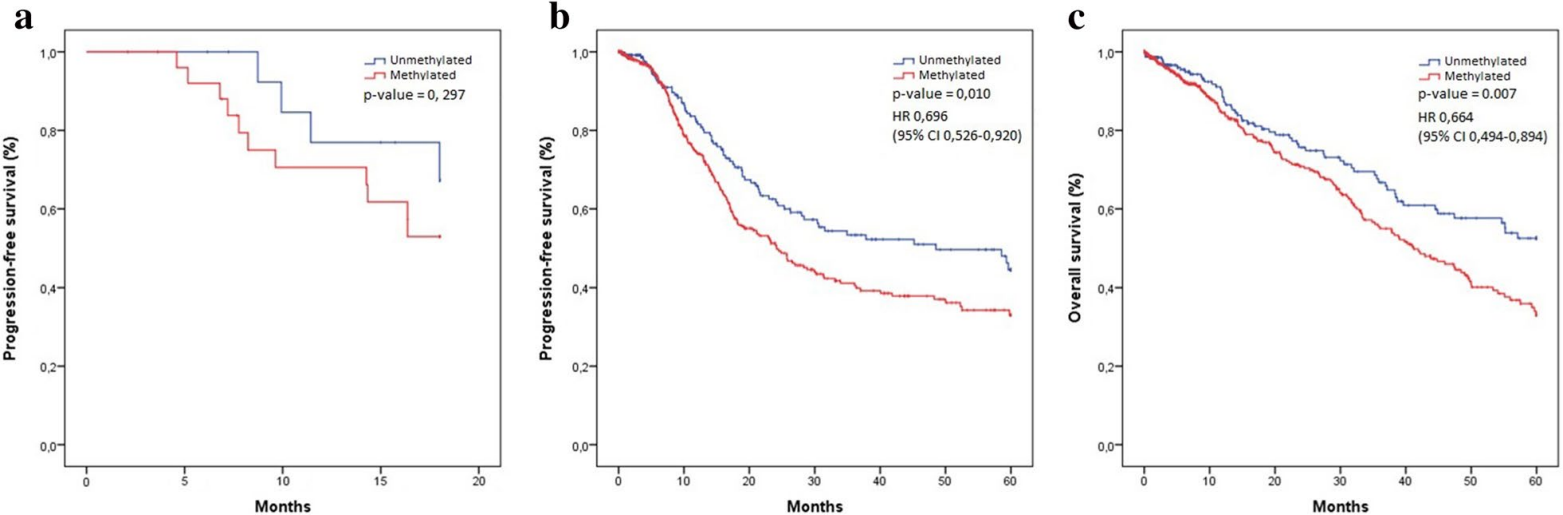
Kaplan–Meier survival curves for early-stage NSCLC in relation to miR-7 methylation. X-axis displays the number of months from diagnosis to progression (**a**, **b**) or death (**c**). Y-axis: Probability of PFS (**a**, **b**) or OS (**c**). **a** Kaplan–Meier survival curve for PFS for MPC cohort (n = 42). The data showed a trend toward increased progression in those with methylated miR-7, but not statistically significant (p = 0.297). **b** Kaplan–Meier survival curve for PFS for TCGA cohort (n = 795). The PFS of patients with methylated miR-7 was significantly lower (p = 0.010, HR = 0.696). **c** Kaplan–Meier survival curve for OS for TCGA cohort (n = 879). The graph showed a trend toward increased mortality in miR-7 methylated group. Methylated mirR-7 demonstrated a significantly shorter survival when compared to the patients with unmethylated miR-7 (p = 0.007, HR = 0.894)

Attending to the statistical significant observed between miR-7 methylation levels and tumor histologic type we wanted to confirm its clinical significance. Because the number of unmethylated adenocarcinoma patients was only 23 (5%), we obtained almost a statistical significance between methylated and unmethylated adenocarcinoma patients (p = 0.064). However, when we segregate patients regarding methylation status, the difference in PFS between the miR-7 methylated patients with adenocarcinoma and miR-7 methylated patients with squamous histology was statistically significant (median 18.9 versus 59.7 months, p = 0.002). Therefore, miR-7 methylation could be used as a bad prognosis biomarker, especially for adenocarcinoma patients (Fig. 3b).

**Fig. 3:**
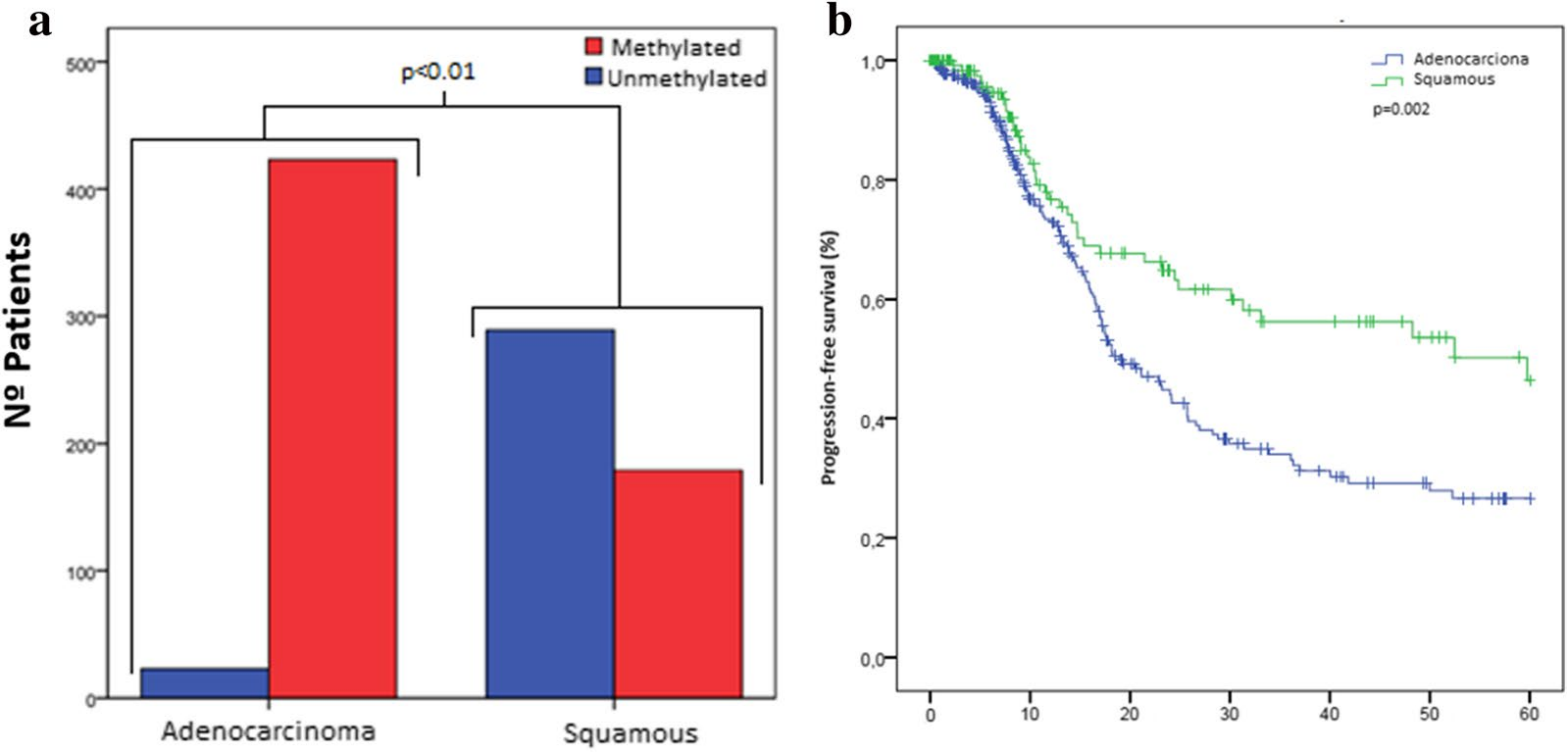
Kaplan–Meier analysis showing the PFS of adenocarcinoma and squamous carcinoma in patients with methylated NSCLC. (**a**) Representation of miR-7 methylation status among adenocarcinoma and squamous tumor histologic types, showing that the presence of methylation is more common in adenocarcinoma than in squamous tumor types. (**b**) Kaplan–Meier survival curve for PFS for TCGA early-stages NSCLC patients harboring miR-7 methylation (n = 523). 370 patients with adenocarcinoma and 153 squamous cell carcinoma. The PFS of patients with adenocarcinoma was significantly lower (p = 0.002)

In advanced stages NSCLC patients, no statistically differences in PFS or OS were found in any of the two cohorts analyzed, probably due to the data distribution since most patients were methylated (Additional file 1; Fig. S2) .

Next, to study the relationship between miR-7 methylation and cisplatin-response treatment in the 31 metastatic NSCLC patients, we checked their radiological study according to RECIST criteria v1.1. Bearing in mind the results of radiological evaluation, treatment response was classified in partial response (PR), stable disease (SD) and progressive disease (PD) and compared with the miR-7 methylation status of the samples. We found that all the patients (100%) with progressive disease harbored miR-7 methylated (Table 2). We observed a high methylation ratio that supports the baseline methylation level found in DNA from non-neoplastic tissue. These data also indicate that miR-7 probably is involved in the early establishment of lung tumorogenesis. Our limited sample size unable a strong statistical significance in terms of RECIST and unfortunately the RECIST data is not recruited in the TCGA database. The sample size for advanced stages is also limited, but results strongly indicate that miR-7 methylation is a bad prognostic factor in terms of OS and PFS in early stages NSCLC. In fact, this results may indicate that methylation of miR-7 is an early event previous to the carcinogenesis and explain that the clinical value of this potential marker does not associate with advanced stages but could be of great interested to characterize patients at early-stages.

We found that miR-7 unmethylation in the lung tumor samples were associated with better PFS and OS in the two analyzed cohorts, supporting their role previously published in ovarian cancer by our group. In addition it seems to have an additional value of poor prognosis for early-stages adenocarcinoma patients harboring miR-7 methylated. One major challenge in early diagnosis in NSCLC is to identify the subgroup of patients that could benefit for adjuvant therapy and our data stablish the basis for epigenetic classification on early-stage NSCLC that could influence treatment decisions in the future.

In conclusion, our results show that the presence of miR-7 methylation at early-stage NSCLC is suggestive of a tumor aggressive behavior, especially for adenocarcinoma patients, and presents the evaluation of miR-7 methylation as a novel tool for clinical use in the stratification of NSCLC patients.

## Additional file

**Additional file 1. Figure S1.** Example of methylated and unmethylated amplification by qMSP. **Figure S2.** Kaplan-Meier survival curves for advanced-stage NSCLC in relation to miR-7 methylation.

## Data Availability

Not applicable.

## Abbreviations

NSCLC: non small cell lung cancer
FFPE: Formalin-Fixed Paraffin embedded
PMC: La Paz University Hospital, Hospital del Mar, and CIBERES
qMSP: quantitative Methylation-Specific PCR
TCGA: The Cancer Genome Atlas
PFS: progression-free survival
OS: overall survival
