# Supplementary material for: miR-7 methylation as a biomarker to predict poor survival in early-stage non-small cell lung cancer patients
Source: Cell Biosci. 2019 Aug 7;9:63. doi: 10.1186/s13578-019-0326-7 (PMC6686393; doi:10.1186/s13578-019-0326-7)
Supplement: Supplementary file 1 — Additional file 1. Figure S1. Example of methylated and unmethylated amplification by qMSP. Figure S2. Kaplan-Meier survival curves for advanced-stage NSCLC in relation to miR-7 methylation. [file 13578_2019_326_MOESM1_ESM.pdf]

Additional

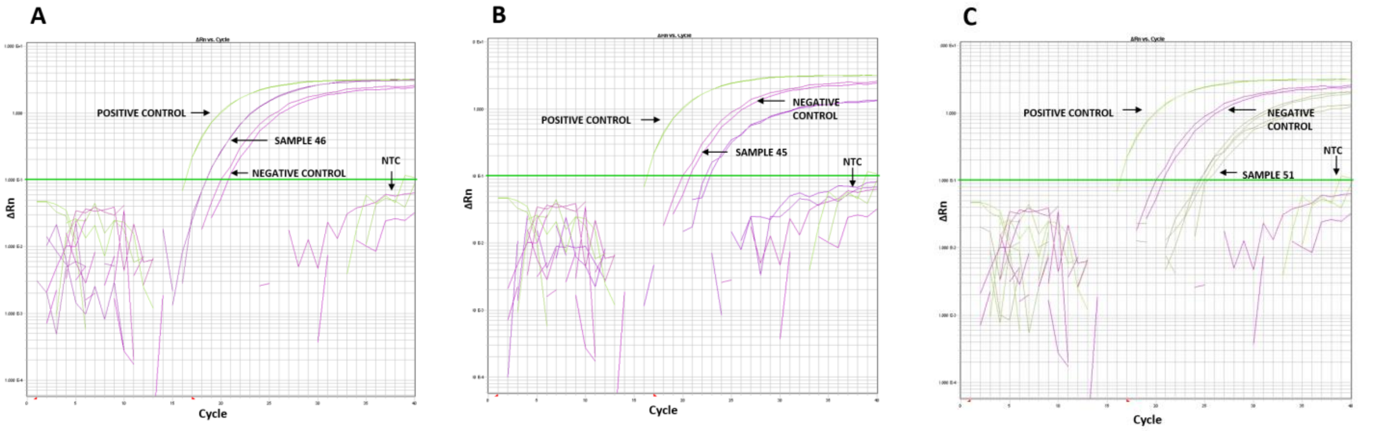

SAMPLE	PROBE (FAM =methylated VIC=unmethylated)	CYCLE
46	FAM	18.44
46	FAM	18.44
46	VIC	Not amplified
46	VIC	Not amplified
POSITIVE CONTROL	FAM	16.23
POSITIVE CONTROL	FAM	16.25
POSITIVE CONTROL	VIC	Not amplified
POSITIVE CONTROL	VIC	Not amplified
NEGATIVE CONTROL	FAM	Not amplified
NEGATIVE CONTROL	FAM	Not amplified
NEGATIVE CONTROL	VIC	20.69
NEGATIVE CONTROL	VIC	20.14
NTC	FAM	Not amplified
NTC	VIC	Not amplified

SAMPLE	PROBE (FAM =methylated VIC=unmethylated)	SAMPLE
45	FAM	Not amplified
45	FAM	Not amplified
45	VIC	22.74
45	VIC	22.43
POSITIVE CONTROL	FAM	16.23
POSITIVE CONTROL	FAM	16.25
POSITIVE CONTROL	VIC	Not amplified
POSITIVE CONTROL	VIC	Not amplified
NEGATIVE CONTROL	FAM	Not amplified
NEGATIVE CONTROL	FAM	Not amplified
NEGATIVE CONTROL	VIC	20.69
NEGATIVE CONTROL	VIC	20.14
NTC	FAM	Not amplified
NTC	VIC	Not amplified

SAMPLE	PROBE (FAM =methylated VIC=unmethylated)	CYCLE
51	FAM	24.48
51	FAM	24.92
51	VIC	24.34
51	VIC	25.51
POSITIVE CONTROL	FAM	16.23
POSITIVE CONTROL	FAM	16.25
POSITIVE CONTROL	VIC	Not amplified
POSITIVE CONTROL	VIC	Not amplified
NEGATIVE CONTROL	FAM	Not amplified
NEGATIVE CONTROL	FAM	Not amplified
NEGATIVE CONTROL	VIC	20.69
NEGATIVE CONTROL	VIC	20.14
NTC	FAM	Not amplified
NTC	VIC	Not amplified

Additional File Figure S1: Example of methylated and unmethylated amplification by qMSP.

Positive control (IVD, in vitro methylated DNA). Negative control (DNA known to be 100% unmethylated). NTC (No Template Control). **A.** In sample 46 only FAM probe amplified. Thus, sample 46 is a methylated sample (100% methylated). **B**. In sample 45 only VIC probe amplified. Thus, sample 46 is a methylated simple (0 % methylated). **C.** In sample 51 both probes amplified. Thus, sample 51 is a methylated simple (53.9 % methylated)

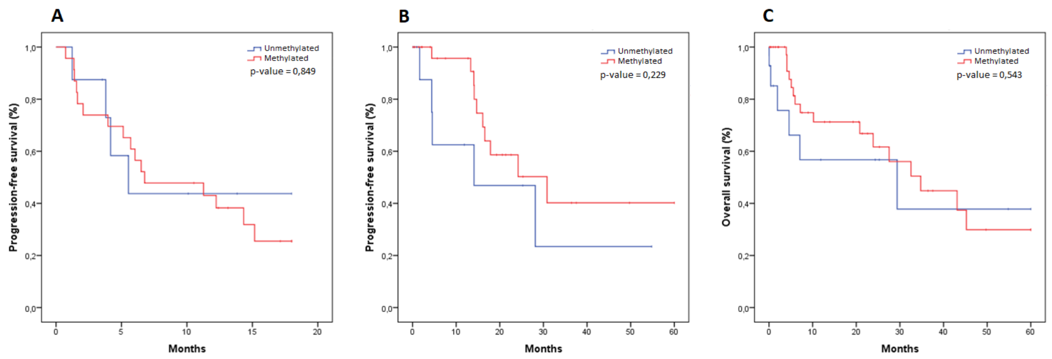

Additional File Figure S2. Kaplan-Meier survival curves for advanced-stage NSCLC in relation to miR-7 methylation.

X-axis displays the number of months from diagnosis to progression (A and B) or death (C). Y-axis: Probability of PFS (A and B) or OS (C). **A.** Kaplan–Meier survival curve for PFS for MPC cohort (n=31). Analysis showed no significant difference between methylated and unmethylated mirR-7 groups (p=0,849). **B.** Kaplan–Meier survival curve for PFS for TCGA cohort (n=40). Methylated and unmethylated miR-7 group were not significantly different (p=0.229). **C.** Kaplan–Meier survival curve for OS for TCGA cohort (n=55). No significant differences in survival rates were found among methylated and unmethylated group (p=0.543)
